# CRISPR/Cas9 deletion of ORMDLs reveals complexity in sphingolipid metabolism

**DOI:** 10.1016/j.jlr.2021.100082

**Published:** 2021-04-30

**Authors:** Christopher D. Green, Cynthia Weigel, Clement Oyeniran, Briana N. James, Deanna Davis, Usha Mahawar, Jason Newton, Binks W. Wattenberg, Michael Maceyka, Sarah Spiegel

**Affiliations:** Department of Biochemistry and Molecular Biology, VCU School of Medicine, Richmond, VA, USA

**Keywords:** Ormdl, serine palmitoyltransferase, ceramide, sphingolipid biosynthesis, sphingosine-1-phosphate, CRISPR/Cas9, sphingomyelin, sphingoid bases, GalCer, galactosylceramides, GluCer, glucosylceramide, gRNA, guide RNA, HDM, house dust mite, PI, propidium iodide, ROS, reactive oxygen species, S1P, sphingosine-1-phosphate, SPL, S1P lyase, SPT, serine palmitoyltransferase

## Abstract

The serine palmitoyltransferase (SPT) complex catalyzes the rate-limiting step in the de novo biosynthesis of ceramides, the precursors of sphingolipids. The mammalian ORMDL isoforms (ORMDL1-3) are negative regulators of SPT. However, the roles of individual ORMDL isoforms are unclear. Using siRNA against individual *ORMDLs*, only single si*ORMDL3* had modest effects on dihydroceramide and ceramide levels, whereas downregulation of all three *ORMDL*s induced more pronounced increases. With the CRISPR/Cas9-based genome-editing strategy, we established stable single *ORMDL3* KO (ORMDL3-KO) and *ORMDL1/2/3* triple-KO (ORMDL-TKO) cell lines to further understand the roles of ORMDL proteins in sphingolipid biosynthesis. While ORMDL3-KO modestly increased dihydroceramide and ceramide levels, ORMDL-TKO cells had dramatic increases in the accumulation of these sphingolipid precursors. SPT activity was increased only in ORMDL-TKO cells. In addition, ORMDL-TKO but not ORMDL3-KO dramatically increased levels of galactosylceramides, glucosylceramides, and lactosylceramides, the elevated N-acyl chain distributions of which broadly correlated with the increases in ceramide species. Surprisingly, although C16:0 is the major sphingomyelin species, it was only increased in ORMDL3-KO, whereas all other N-acyl chain sphingomyelin species were significantly increased in ORMDL-TKO cells. Analysis of sphingoid bases revealed that although sphingosine was only increased 2-fold in ORMDL-TKO cells, levels of dihydrosphingosine, dihydrosphingosine-1-phosphate, and sphingosine-1-phosphate were hugely increased in ORMDL-TKO cells and not in ORMDL3-KO cells. Thus, ORMDL proteins may have a complex, multifaceted role in the biosynthesis and regulation of cellular sphingolipids.

Sphingolipids are a diverse family of essential eukaryotic lipids characterized by the presence of a sphingoid base. Sphingolipids are not only essential structural components of cell membranes, their metabolites have diverse and important roles in intracellular and intercellular signaling (1, 2). For example, sphingosine-1-phosphate (S1P) is a ligand for a family of five specific G protein–coupled receptors (S1PR1-5) and regulates a myriad of physiological and pathophysiological processes important in cancer and inflammation, typically promoting cell growth and motility and inhibiting apoptosis (1). Conversely, ceramide is associated with cell growth inhibition, senescence, and apoptosis (2).

De novo ceramide biosynthesis begins at the ER with the condensation of serine and palmitoyl CoA by serine palmitoyltransferase (SPT). The product of this reaction is rapidly reduced to dihydrosphingosine that is acylated to dihydroceramide, after which a 4–5 double bond is introduced in the sphingoid base, forming ceramide, a central metabolite of all complex sphingolipids including sphingomyelin and glycosphingolipids, which are generated in the Golgi. Degradation of ceramide by ceramidases occurs in multiple organelles, predominantly in lysosomes, to generate sphingosine (3). Sphingosine and dihydrosphingosine can be phosphorylated by two sphingosine kinases. Phosphorylated sphingoid bases can be either dephosphorylated for reincorporation into sphingolipids or irreversibly degraded by S1P lyase (SPL), the sole exit point of sphingolipid metabolism.

Owing to the wide variety of vital cellular roles of sphingolipids, their generation, accumulation, and degradation are tightly regulated. The rate-limiting step in de novo ceramide biosynthesis catalyzed by SPT is a key point of regulation. In the yeast *Saccharomyces cerevisiae*, two homologous proteins, ORM1 and ORM2, are in a complex with SPT and negatively regulate its activity (4). ORM proteins are conserved across kingdoms. However, in mammals, the three homologs, termed ORMDL1-3, do not contain the phosphorylation sites known to control their interactions with SPT (5, 6, 7, 8). Intriguingly, it was recently shown that the sphingolipid metabolite ceramide acts as a feedback inhibitor of SPT through a direct or indirect interaction with ORMDL proteins (9).

Much attention has been focused on the regulation of SPT by ORMDL3, as single-nucleotide polymorphisms of *ORMDL3* have been associated with asthma susceptibility (10, 11, 12, 13), although its role in regulating sphingolipid levels has been controversial (14). For example, it was reported that ceramide levels were not significantly altered in *Ormdl3* KO mice (15, 16). In contrast, other studies found ceramide levels in the serum and liver and, to a lesser extent, in lung tissue were increased in *Ormdl3* KO mice (17). Similarly, *Ormdl3*, but not *Ormdl1* or *Ormdl2*, single-KO mice exhibited significantly increased levels of ceramide in the brain (18). Importantly, although all combinations of double *Ormdl* KO mice were viable, triple *Ormdl1/2/3* KOs were embryonic lethal (18). It was suggested that the absence of negative regulation of sphingolipid biosynthesis due to the absence of all ORMDLs in the triple-KO mice could have interrupted critical sphingolipid-regulated developmental processes (18).

Surprisingly, while several mouse studies substantiated a role of ORMDL3 in asthma pathogenesis (19, 20, 21, 22), another study demonstrated that although knockdown of *Ormdl3* increased systemic ceramide levels, it did not alter experimental asthma (17). Moreover, downregulation of *ORMDL3*, but not *ORMDL1* or *ORMDL2*, by siRNA in HepG2 liver cells increased the ceramide precursors dihydrosphingosine and dihydroceramide primarily from de novo biosynthesis (23). Nevertheless, SPT activity was not changed in cells in which *ORMDL3* was deleted or overexpressed (16), and it was suggested that all three *ORMDLs* must be downregulated to relieve the inhibition of SPT (7). Thus, the role of ORMDLs in regulating sphingolipid biosynthesis may go beyond regulation of SPT activity. In this work, we used the CRISPR/Cas9-based genome-editing strategy to generate control, single *ORMDL3* KO, and *ORMDL1/2/3* triple KO cell lines in A549 cells, a model for the study of alveolar type II pulmonary epithelial cells. Our results suggest that ORMDL proteins are partially redundant and their functions are part of a complex and compartmentalized form of regulation necessary for maintaining sphingolipid homeostasis.

## Materials and methods

### Cell culture and downregulation of ORMDLs

A549 human lung carcinoma cells (ATCC, Manassas, VA) were cultured in high-glucose Dulbecco modified Eagle's medium (DMEM, Life Technologies, Carlsbad, CA) containing 10% FBS, 1 mM sodium pyruvate, 2 mM GlutaMAX, and 100 U/ml penicillin-streptomycin (Gibco, Gaithersburg, MD). Cells were transfected with ON-TARGETplus SMARTpool siRNA oligonucleotides (Dharmacon, Lafayette, CO) for human *ORMDL1* (catalog number L-018403-01), *ORMDL2* (catalog number L-017035-02), and *ORMDL3* (catalog number L-01002-02) or scrambled siRNA (siControl) using the Lipofectamine RNAiMAX transfection reagent (Life Technologies).

### Quantitative real-time PCR

Total RNA was prepared with TRIzol and reverse-transcribed using the high-capacity cDNA Archive kit (Life Technologies). Premixed primer-probe sets and TaqMan Universal PCR Master Mix (Life Technologies) were used to determine mRNA levels. cDNAs were amplified using the CFX Connect real-time PCR detection system (Bio-Rad). Gene expression levels were calculated with the ΔΔCt method, normalized to *GAPDH* expression.

### Generation of *ORMDL* KO A549 cells with CRISPR/Cas9

*ORMDL* KO A549 cells were generated by lentiviral delivery of Cas9 and target-specific guide RNAs (gRNAs). Oligos encoding the gRNAs for *ORMDL* isoforms 1, 2, and 3, as well as a nontargeting control, were selected from a previously reported library (24) and cloned into lentiCRISPR.v2, kindly provided by Feng Zhang (Addgene plasmid # 52961; RRID: Addgene 52961 (25)). The following single gRNA sequences were used for gRNA control and to target specific *ORMDL* genes: *ORMDL1* sgRNA, ACCCGTGTCATGAACAGCCG; *ORMDL2* sgRNA, ACCCGAGTGATGAATAGCCG; *ORMDL3* sgRNA, CGAGGTGAACCCCAACACGC; control sgRNA, CGAGGTGAACCCCAACACGC targeted to *ORMDL3*, which did not cleave (CTL1), or nontarget control sgRNA, AAAAAGCTTCCGCCTGATGG (CTL2). Lentiviruses were then generated by transient transfection of the viral plasmid and packaging plasmids into Lenti-X 293T cells (Takara). A549 cells were transduced with lentiviruses in the presence of polybrene (8 μg/ml). 72 h after infection, cells were selected under 1 μg/ml puromycin for 7 days. The surviving cells were then separated as single cells into a 96-well plate by fluorescence-activated cell sorting and subjected to puromycin selection for another 7 days. Individual clones were expanded, and ORMDL protein expression was examined by immunoblotting. See supplemental Table S1 for details.

Gene editing of clones with successful reduction of ORMDL protein expression was validated by PCR using Phusion High-Fidelity DNA Polymerase (New England Biolabs) and primers designed for ~500 bp regions encompassing *ORMDL* gRNA target-specific loci and off-target loci predicted by CRISPOR (26). PCR products were sequenced and analyzed for gene editing using CRISP-ID (27). No off-target editing of sequences predicted to have the highest probability of such an effect was detected (supplemental Table S2).

### Cell proliferation assays

Cell proliferation was determined with WST-8 [2-(2-methoxy-4-nitrophenyl)-3-(4-nitrophenyl)-5-(2,4-disulfophenyl)-2H-tetrazolium, monosodium salt] using the Cell Counting Kit-8 (CK04, Dojindo Molecular Technologies) as described previously (28).

### Viability and live-dead cell assays

Cells were seeded in black 96-well plates with clear bottoms (Greiner, Frickenhausen, Germany) at a cell density of 5,000 cells per well in 200 μl DMEM supplemented with 10% FBS, 1 mM sodium pyruvate, and 2 mM GlutaMAX. After 24 h, cells were stimulated with house dust mite (HDM) from Greer Laboratories (catalog no. XPB70D3A2.5; Lenoir, NC).

Cell viability was determined after 20 h with the Deep Blue Cell Viability Kit (BioLegend, San Diego, CA). The reduction of resazurin to resorufin was measured after 4 h incubation with a TECAN Infinite M1000 Pro fluorescence plate reader (Männedorf, Switzerland) at excitation and emission wavelengths of 550 nm and 610 nm, respectively.

For live/dead cell assays, 24 h after treatments, plates were centrifuged at 300 *g* for 5 min at room temperature, and 100 μl of the media was removed and replaced by 100 μl of HBSS containing 3 μM calcein-AM (BioLegend, San Diego, CA) and 5 μM propidium iodide (PI). After 30 min of incubation at 37°C, PI fluorescence was measured with a TECAN Infinite M1000 Pro fluorescence plate reader at 530 nm/620 nm. Plates were washed and refilled with 200 μl of HBSS per well, and calcein fluorescence was measured at 485 nm/535 nm.

### Permeability assays

FITC-dextran permeability assays were carried out as described previously (29), with minor modifications. Briefly, 50,000 cells in 200 μl DMEM supplemented with 10% FBS, 1 mM sodium pyruvate, and 2 mM GlutaMAX were seeded in the upper chamber of a 24-well tissue culture plate with transwell cell culture inserts (0.4 μm pore size, Sarstedt, Nürnbrecht, Germany). The lower chamber contained 600 μl of culture media. Cells were cultured until confluency. The medium in the lower chamber was then replaced, and the medium in the upper chamber was replaced with 200 μl of media without or with 100 μg/ml HDM and 2 mg/ml of 70 kDa FITC-dextran (Sigma-Aldrich, Steinheim, Germany). After 24 h, FITC-dextran in the lower chambers was measured. Media from the lower chambers were transferred into black 96-well plates with clear bottoms, and fluorescence intensity was measured with a TECAN Infinite M1000 Pro fluorescence plate reader (Tecan, Männedorf, Swiss) at 485/530 nm.

### Measurement of ROS levels

Reactive oxygen species (ROS) levels were measured as previously described (30).

### Sphingolipid analyses

Sphingolipids were measured as described previously (23). Briefly, cells were seeded in 6-well plates at 350,000 cells per well. Cells were allowed to attach for 18 h, washed three times in ice-cold PBS, and then scraped in ice-cold PBS plus Halt protease and phosphatase inhibitors (Thermo Fisher). Samples were added to 1 ml ice-cold methanol, internal standards added, sphingolipids extracted, and subsequently quantified by LC-ESI-MS/MS (5500 QTRAP, ABI). Glucosylceramides (GluCer) and galactosylceramides (GalCer) were separated and analyzed using a LC-Si column (Supelco 2.1 × 250 mm LC-Si) as described previously (31). Sphingolipid levels were determined as pmol/mg protein. Results shown are cumulative data from three independent experiments (three different cell passage numbers) each with three biological replicates (three different wells of cells).

### Western blotting

Proteins were measured with the Pierce BCA Protein Assay Kit (Thermo Scientific; Rockford, IL), and equal amounts were separated by SDS-PAGE and transferred onto 0.45 μm nitrocellulose. Blots were incubated with the following primary antibodies: anti-ORMDL3 (1:1,000; Millipore Corp, Billerica, MA); anti-SPTLC1 (1:1,000; BD Biosciences, San Jose, CA); GAPDH (#2118, 1:5,000; Cell Signaling Technology, Danvers, MA); and anti-SPL (1:1,000, Clone: H-300 Santa Cruz Biotechnology) and then incubated with secondary antibodies conjugated with horseradish peroxidase (goat anti-rabbit; 1:5,000; Jackson ImmunoResearch, West Grove, PA). Immunopositive bands were visualized using SuperSignal West Pico Stable Peroxide Solution or Dura Extended Duration Substrate (Thermo Scientific). Bands were quantified with ImageJ software and normalized to GAPDH loading controls.

### SPT activity in intact cells

A549 CRISPR/Cas9 clones were plated in 24-well plates at 7 × 10^4^ cells/well in complete DMEM. 24 h after plating, media were removed and cells washed with PBS. SPT activity in cells was measured as described (9). Briefly, serine-free media containing 10 μCi/ml ^3^H-serine were added (200 μl/well). Cells were labeled for 60 min, washed once with PBS, and 200 μl/well of PBS added. As a negative control, the SPT inhibitor myriocin (1 μM) was added during the 60 min labeling period. Cells were extracted with 400 μl/well alkaline methanol (MeOH + 0.7 g/100 ml KOH). The extracts were transferred to 2 ml screw cap tubes, and total sphingolipids were extracted with 100 μl CHCl_3_ by vortexing and brief centrifugation. 500 μl CHCl_3_ was added followed by 300 μl alkaline methanol. After mixing and centrifugation, the upper, aqueous phases were removed and the lower organic phases were washed twice with 1 ml alkaline water (100 μl of 2 N NH_3_OH). 400 μl aliquots of the organic phase were dried in scintillation vials and counted (BetaMax ES, MP Biomedicals, Solon, OH). ^3^H incorporated into sphingolipids was normalized to total protein.

### SPL activity

SPL activity in cell lysates was measured essentially as described (32). Briefly, cells were seeded onto 10 cm^2^ plates, allowed to attach overnight, washed three times with cold PBS, scraped in 100 μl 0.5 M potassium phosphate buffer (pH 7.4), and lysed by brief sonication and repeated freeze-thaw cycles. Aliquots (75 μl) were then added to 96-well plates with 15 μl of the reaction buffer (0.5 M) potassium phosphate (pH 7.4), containing 25 μM Na_3_VO_4_, 0.25 mM pyridoxal 5`-phosphate, and 125 μM SPL fluorogenic substrate (Cayman Chemical, Ann Arbor, MI). After incubation for 6 h at 37°C in the dark, reactions were stopped with 50 μl of methanol and incubated for 2 h. Fluorescence was measured (360 nm excitation/465 nm emission) with a TECAN Infinite M1000 fluorescent plate reader. Data are expressed as relative fluorescence units normalized to total protein determined colorimetrically with bicinchoninic acid (32).

### Statistical analyses

Statistical significance was determined by unpaired two-tailed Student's *t* test for comparison of two groups and one-way ANOVA with post hoc comparison for experiments consisting of three or more groups (GraphPad Prism; GraphPad Software, La Jolla, CA). *P* ≤ 0.05 was considered significant. All experiments were repeated independently at least three times.

## Results

### Effects of ORMDL knockdown on ceramide levels

**Fig. 1:**
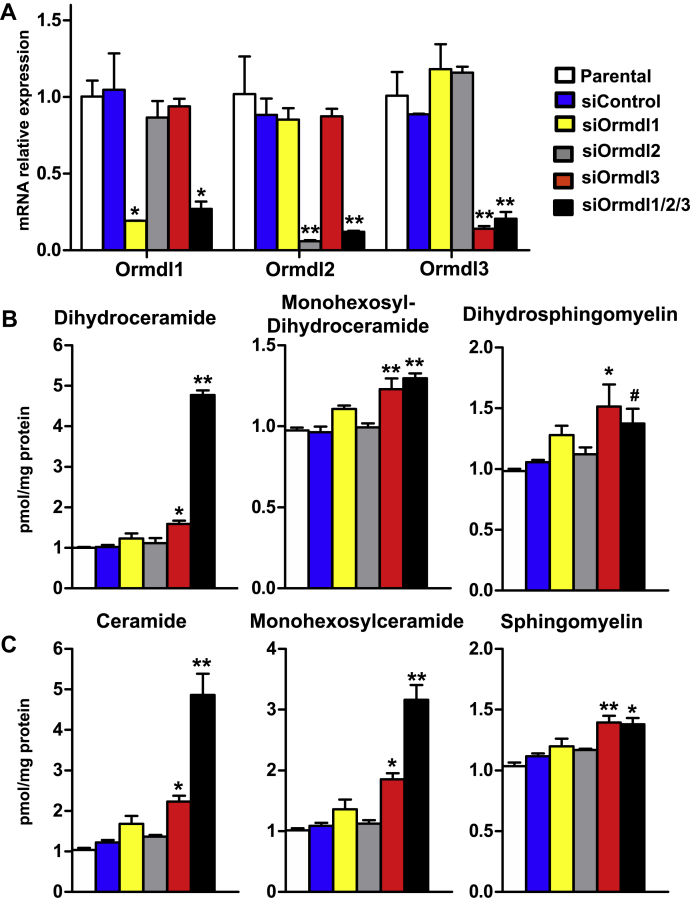
Effect of individual and triple siRNA *ORMDL* knockdown on ceramide levels. A549 cells were transfected with scrambled siRNA (siControl), specific siRNAs for individual *ORMDL* isoforms, or for all three isoforms, as indicated. A: mRNA levels of individual *ORMDL* isoforms were determined by quantitative PCR and normalized to *GAPDH*. n = 3 biological replicates. B and C: Sphingolipids were extracted and total levels of dihydroceramide, monohexosyldihydroceramide, and dihydrosphingomyelin and (B) total levels of ceramide, monohexosylceramide, and sphingomyelin (C) were determined by LC-ESI-MS/MS. Data are expressed as fold increase compared with parental and are the means ± SEM (n = 9). Each data point represents three independent experiments each with three separate biological replicates. ^#^*P* < 0.05; ∗*P* < 0.01; ∗∗*P* < 0.001 compared with siControl (A) or parental (B and C). Statistical analysis by two-way ANOVA (A) and one-way ANOVA with Dunnett’s post hoc test (B and C).

Given previous results with ORMDL3 regulation of ceramide levels in epithelial cells (21) together with the recent observation in mice that ORMDL3 controls brain sphingolipids (18), we sought to compare the effect of downregulating individual ORMDLs with that of ORMDL1/2/3 triple knockdown in A549 cells, which express all three isoforms, on dihydroceramide, an intermediate formed solely in the de novo sphingolipid biosynthetic pathway, and on ceramide that can be generated both by de novo and recycling/salvage pathways. Expression of each of the individual *ORMDL*s was efficiently downregulated with specific siRNAs by 80% without significantly affecting the expression of the other two (Fig. 1A). Simultaneous knockdown of all three *ORMDL*s decreased mRNA levels to a similar extent as the individual siRNAs alone (Fig. 1A). In agreement with previous reports (18, 23), downregulation of *ORMDL1* or *ORMDL2* alone had no effect on dihydroceramide, nor did they significantly change levels of ceramide containing sphingolipids (Fig. 1B, C). In contrast, *ORMDL3* knockdown modestly but significantly increased dihydroceramide, as well as monohexosyldihydroceramide and dihydrosphingomyelin (Fig. 1B), consistent with a role in regulating de novo synthesis. Moreover, knockdown of *ORMDL3* induced elevations in ceramide and the ceramide-containing sphingolipids, monohexosylceramide, and sphingomyelin (Fig. 1C). Knockdown of all three *ORMDL* isoforms led to a dramatic 5-fold increase in dihydroceramide and ceramide, which was greater than the induction by knockdown of *ORMDL3* alone (Fig. 1B, C). However, the triple knockdown only increased monohexosylcermide levels above that of si*ORMDL3* alone, while the levels of dihydrosphingomyelin and sphingomyelin were similarly increased (Fig. 1B, C).

### Generation of ORMDL1/2/3 triple KO human lung epithelial cells

Given that downregulation of *ORMDL3*, but not *ORMDL1* or *ORMDL2*, increased levels of sphingolipids and that downregulation of all three *ORMDL*s dramatically increases sphingolipids (Fig. 1) together with previous observations that although *Ormdl* double KO mice are viable but the triple KO is embryonic lethal (18), it was important to establish A549 cells with stable *ORMDL3* KO and triple *ORMDL1/2/3* KOs to investigate their effects on biosynthesis and levels of sphingolipids and examine functional redundancy of the *ORMDLs*. CRISPR-Cas9–based genome editing, which allows modifications to genomes with a precision and efficiency unmatched by previous technologies (25, 33), was used to generate single *ORMDL3* KO (ORMDL3-KO1 and ORMDL3-KO2) clones, an ORMDL1/2/3 triple KO (ORMDL-TKO) clone, and two control (CTL1 and CTL2) clones (supplemental Table S1). These clones were validated by sequencing the targeted *ORMDL* cleavage loci using PCR products of primers designed for ~500 bp regions encompassing the *ORMDL* gRNA target-specific loci and predicted off-targets (supplemental Fig. S1 and supplemental Tables S1 and S2).

**Fig. 2:**
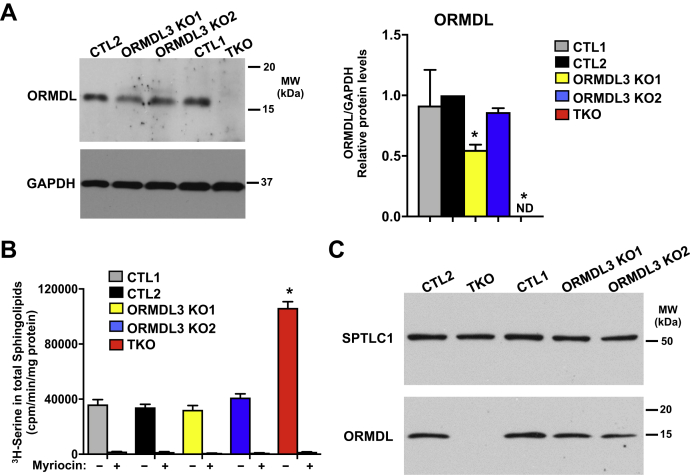
CRISPR/Cas9 deletion of all three ORMDL isoforms increases SPT activity. A: ORMDL protein levels in A549 cells with CRISPR/Cas9-mediated KO of ORMDL3 alone in two clones (ORMDL3-KO1 and ORMDL3-KO2) or in KO of all three ORMDL3 isoforms (ORMDL3-TKO) as determined by Western blotting compared with control (CTL1 and 2). (n= 4). B: SPT activity was determined by incorporation of [3H]serine into de novo–synthesized sphingolipids in the indicated clones. The SPT inhibitor myriocin was used as a negative control. Data are the means ± SEM (n = 6). Each data point represents two independent experiments each with three separate biological replicates. ∗*P* < 0.01 compared to CTL. Statistical analysis by one-way ANOVA with Tukey’s post hoc test. C: Duplicate samples were analyzed by Western blotting with the indicated antibodies to demonstrate equal levels of the SPTLC1 subunit of the SPT complex. ND, not detected; SPT, serine palmitoyltransferase.

Immunoblotting with an antibody that recognizes all three ORMDL isoforms showed that the ORMDL3-KO1 cells had modestly reduced levels of ORMDL proteins, suggesting that ORMDL3 was absent and that ORMDL1 and ORMDL2 were not upregulated at the protein level (Fig. 2A). Moreover, ORMDL proteins were not detected in the ORMDL-TKO cells. The effect of ORMDL deletions on in situ SPT activity was assessed by measurement of incorporation of ^3^H-serine into sphingolipids (9). A dramatic increase in SPT activity was only observed in ORMDL-TKO cells (Fig. 2B). There were no changes in the level of the SPTLC1 subunit of SPT after deletion of all ORMDL isoforms (Fig. 2C), as ORMDL proteins are allosteric regulators of SPT. Because there were no differences between the two CTLs and ORMDL3-KO1 had reduction in ORMDL3 protein level, CTL1 and ORMDL3-KO1 cells were selected for further studies together with ORMDL-TKO cells.

### Deletion of all three ORMDLs potentiates increases in dihydroceramides and ceramides induced by ORMDL3 KO

**Fig. 3:**
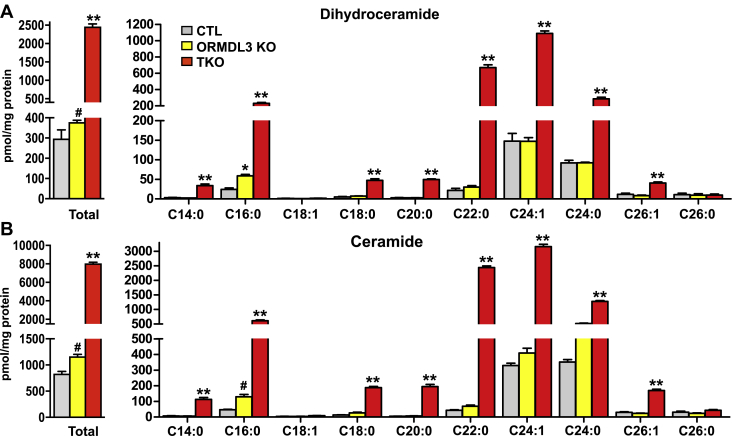
Deletion of all three ORMDLs potentiates increases in dihydroceramides and ceramides induced by deletion of ORMDL3. Sphingolipids were extracted from control (CTL1), *ORMDL3* single KO (ORMDL3 KO1), and triple KO *ORMDL1,2,3* (TKO) cells and total (A) dihydroceramides and (B) ceramides and their acyl chain species were measured by LC-ESI-MS/MS. Numbers indicate the chain length followed by the number of double bonds in the fatty acid. Data are the means ± SEM (n = 9). Each data point represents three independent experiments each with three separate biological replicates. ^#^*P* < 0.05; ∗*P* < 0.01; ∗∗*P* < 0.001 compared with CTL by one-way ANOVA with Tukey’s post hoc analysis for each species.

De novo synthesis of ceramide on the ER proceeds from the formation of dihydrosphingosine that is subsequently acylated with fatty acids from 14 to 26 carbons long to form dihydroceramides. Then, a 4–5 double bond is introduced in the sphingoid base to generate ceramide. Consistent with previous results with an siRNA approach (21), deletion of ORMDL3 alone led to small but significant increases in both total dihydroceramides and ceramides and several of their acyl chain species (Fig. 3A, B and supplemental Table S3). Elevation of both dihydroceramides and ceramides was greatly increased further by deletion of all three ORMDL isoforms in the ORMDL-TKO cells by 6.5- and 6.9-fold, respectively (Fig. 3A, B). The results are consistent with redundant function of ORMDL isoforms to regulate SPT activity.

Although deletion of all three ORMDLs caused a very large increase in ceramide that has been associated with cell growth retardation and cell death (2), cellular proliferation was only slightly reduced in these cells, whereas deletion of *ORMDL3* alone slightly increased proliferation (supplemental Fig. S2A). We previously showed that the allergen HDM induces cell death and ROS in lung epithelial cells in a ceramide-dependent manner (30). Treatment with HDM, as expected, reduced viability (supplemental Fig. S2B) and increased cell death determined by live-dead double staining with calcein-AM and red-fluorescent PI (supplemental Fig. S2C). However, there were no major differences in these HDM responses of either ORMDL-TKO or ORMDL3 KO cells compared with control cells. Likewise, HDM-induced ROS generation was not affected by deletion of either ORMDL1,2,3 or ORMDL3 alone (supplemental Fig. S2D). Similarly, HDM-induced permeability of these cells to FITC-dextran was also the same (supplemental Fig. S2E).

### Triple *ORMDL1/2/3* but not *ORMDL3* single KO dramatically increases monoglycosylceramides

**Fig. 4:**
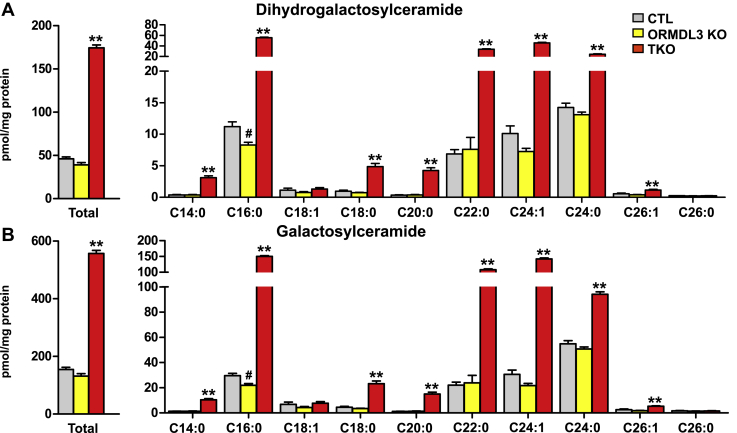
Triple ORMDL1/2/3 but not ORMDL3 single KO increases galactosylceramides. Sphingolipids were extracted from the indicated cells and levels of total (A) dihydrogalactosylceramide and (B) galactosylceramide and their acyl chain species were measured by specific LC-ESI-MS/MS to separate monohexosylceramides. The numbers indicate the chain length followed by the number of double bonds in the fatty acid. Data are the means ± SEM (n = 9). Each data point represents three independent experiments each with three separate biological replicates. ^#^*P* < 0.05; ∗*P* < 0.01; ∗∗*P* < 0.001 compared with CTL by one-way ANOVA with Tukey’s post hoc analysis for each species.

**Fig. 5:**
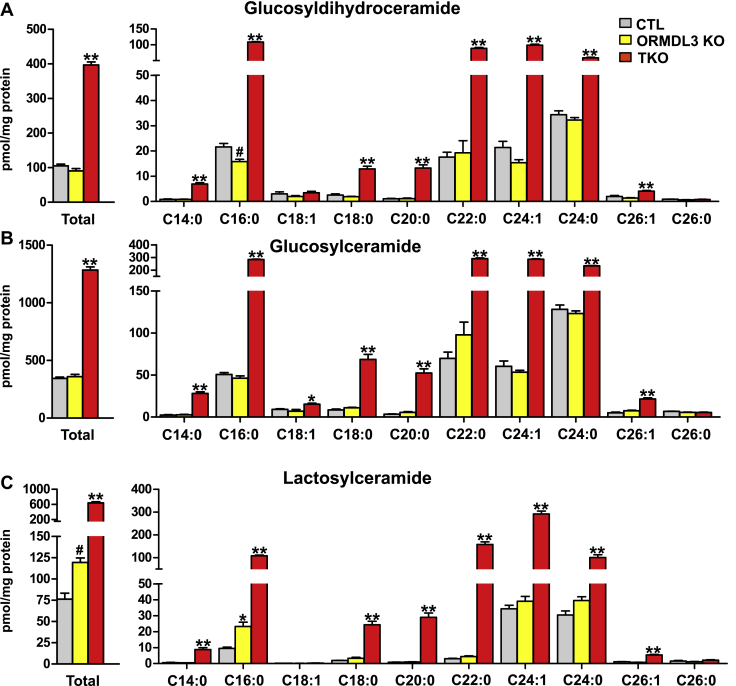
Changes in levels of glucosylceramide and lactosylceramide by ORMDL deletion. Sphingolipids were extracted from the indicated cells, and levels of total (A) dihydroglucosylceramide, (B) glucosylceramide, and (C) lactosylceramide and their acyl chain species were measured by LC-ESI-MS/MS. The numbers indicate the chain length followed by the number of double bonds in the fatty acid. Data are the means ± SEM (n = 9). Each data point represents three independent experiments each with three separate biological replicates. ^#^*P* < 0.05; ∗*P* < 0.01; ∗∗*P* < 0.001 compared with CTL by one-way ANOVA with Tukey’s post hoc analysis for each species.

The synthesis of glycosphingolipids is initiated by the transfer of either galactose to ceramide at the lumen of the ER forming GalCer or glucose to ceramide at the *cis*-Golgi forming GluCer. Given the different locations of where these monohexoses are added to the primary hydroxyl of ceramide, we next measured their levels by another LC-ESI-MS/MS run with a longer silica column that separates these isomers. Deletion of *ORMDL3* alone did not affect galactosyl dihydroceramides or GalCer (Fig. 4A, B), nor did it have major effects on levels of glucosyl dihydroceramides or GluCer (Fig. 5A, B). In contrast, in triple *ORMDL1/2/3* KO cells, there were large increases in all monoglucosylated ceramides, collectively known as cerebrosides (Figs. 4A, B and 5A, B). There were also significant increases in all species of these glycosphingolipids. Moreover, their elevated acyl chain distributions broadly matched the increased acyl chain ceramide species (Fig. 3).

### Changes in levels of lactosylceramide by ORMDL deletion

GluCer made in the Golgi can be further modified by addition of other sugars to form the large, branched oligosaccharides common to glycosphingolipids. One of the first steps in this process is the addition of galactose to the glucosyl moiety, forming lactosylceramides. Therefore, we asked if the elevations of GluCer observed in the ORMDL-TKO cells (Fig. 5A, B) was reflected by an increase in lactosylceramides. *ORMDL3* deletion alone resulted in small increases in lactosylceramides, particularly in the C16:0 species (Fig. 5C). However, in ORMDL-TKO cells, there were large increases in all lactosylceramide species that was especially pronounced for C20:0 and C22:0 very-long-chain N-acyl chain species (Fig. 5C) and consistent with the acyl chain distribution in ceramides in these cells (Fig. 3). Taken together, these results suggest that a significant amount of the excess ceramides observed in ORMDL-TKO cells can be converted to glycosphingolipids.

### Effects of deletion of ORMDLs on levels of sphingomyelin

**Fig. 6:**
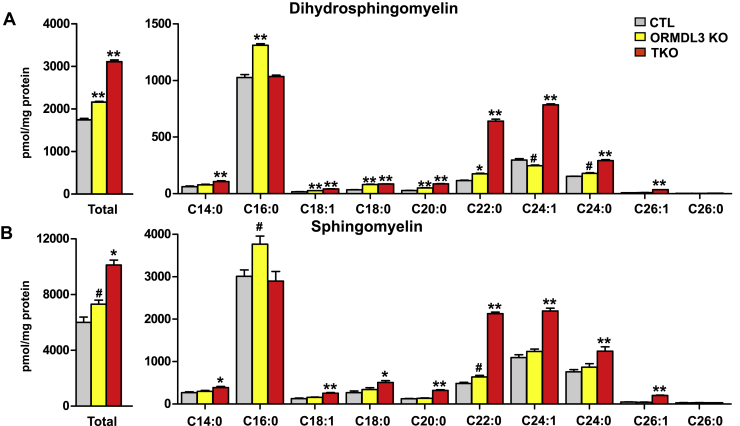
Deletion of all three ORMDLs markedly increases very-long-chain C22:0, C24:1, and C24:0 species but not the C16:0 sphingomyelin species. Sphingolipids were extracted from the indicated cells and levels of total and typical N-acyl chain species of (A) dihydrosphingomyelins and (B) sphingomyelin were measured by LC-ESI-MS/MS. The numbers indicate the chain length followed by the number of double bonds in the fatty acid. Data are the means ± SEM (n = 9). Each data point represents three independent experiments each with three separate biological replicates. ^#^*P* < 0.05; ∗*P* < 0.01; ∗∗*P* < 0.001 compared with CTL by one-way ANOVA with Tukey’s post hoc analysis for each species.

Dihydroceramides and ceramides are also delivered to the Golgi by ceramide transport protein (CERT) through ER-Golgi membrane contact sites for the formation of sphingomyelins (2). We observed that ORMDL3-KO cells had elevated total levels of both dihydrosphingomyelin and sphingomyelin relative to control cells (Fig. 6A, B). Of particular interest is the increase of the major 16:0 species of sphingomyelin. Surprisingly, while total dihydrosphingomyelin and sphingomyelin were further increased in ORMDL-TKO cells, these increases were predominantly in the very-long-chain C22:0, C24:1, and C24:0 species but not in the C16:0 species (Fig. 6A, B).

### Levels of phosphorylated sphingoid bases are dramatically increased in ORMDL-TKO cells

**Fig. 7:**
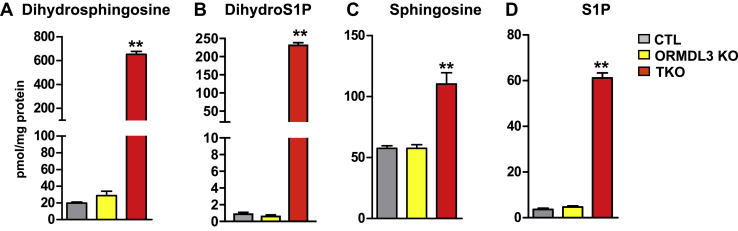
Deletion of all three ORMDL isoforms dramatically increases phosphorylated sphingoid bases. Levels of (A) dihydrosphingosine, (B) dihydro-S1P, (C) sphingosine, and (D) S1P were determined in the indicated cells by LC-ESI-MS/MS. Data are the means ± SEM (n = 9). Each data point represents three independent experiments each with three separate biological replicates. ∗∗*P* < 0.001 compared with CTL by one-way ANOVA with Tukey’s post hoc analysis for each species. S1P, sphingosine-1-phosphate.

Because ORMDL proteins regulate SPT and thus the levels of dihydrosphingosine, the intermediate generated exclusively by de novo sphingolipid biosynthesis, we next examined the effect of ORMDL isoform deletion on its levels. Although deletion of ORMDL3 by itself significantly increased dihydroceramides (Fig. 3A), there was only a small albeit not statistically significant increase in levels of dihydrosphingosine (Fig. 7A). Remarkably, ORMDL-TKO cells had a dramatic, 33-fold increase in dihydrosphingosine levels (Fig. 7A) as might be expected if de novo synthesis was derepressed. Although much of this dihydrosphingosine was converted into dihydroceramide- and dihydroceramide-containing complex sphingolipids (Fig. 3, Fig. 4, Fig. 5, Fig. 6), we found that there was a dramatic, nearly 240-fold increase in dihydro-S1P in ORMDL-TKO cells (Fig. 7B). While levels of sphingosine generated by cleavage of ceramide and ceramide-containing sphingolipids were only doubled in ORMDL-TKO cells, S1P, its phosphorylated form, was increased by nearly 16-fold (Fig. 7C, D).

### Effect of ORMDL3 deletion on SPL

**Fig. 8:**
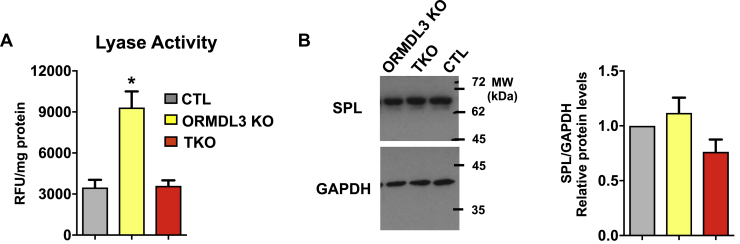
ORMDL3 deletion increases S1P lyase activity. A: SPL activity was measured in lysates from control (CTL1), *ORMDL3* single KO (ORMDL3 KO1), and triple KO *ORMDL1,2,3* (TKO) cells. Data are the mean ± SEM of 2–3 technical replicates from five independent experiments. ∗*P* < 0.05 compared with CTL determined by one-way ANOVA followed by Tukey’s post hoc analysis. B: cell lysates were analyzed by Western blotting with anti-SPL and anti-GAPDH antibody for equal loading and transfer. A representative blot is shown and relative densities of the immunopositive bands normalized to GAPDH quantified. Data are the mean ± SEM of six biological replicates. S1P, sphingosine-1-phosphate; SPL, S1P lyase.

Sphingolipids are degraded after phosphorylation of sphingosine by sphingosine kinases and subsequent irreversible cleavage by ER-localized SPL to ethanolamine phosphate and hexadecanal or hexadecenal from dihydoS1P or S1P, respectively. We were curious whether the dramatic rise in the levels dihydro-S1P and S1P is due to decreased SPL activity in the ORMDL-TKO cells. However, deletion of all ORMDLs had no significant effects on SPL activity as well as protein levels (Fig. 8A, B). This suggests that the accumulation of phosphorylated bases may exceed the ability of SPL to degrade them. Consistent with a recent report suggesting that downregulation of ORMDL3 increases the activity of SPL (34), we found that ORMDL3-KO cells had significantly increased SPL activity that could explain their lack of increased S1P and dihydro-S1P (Fig. 8B, D). Thus, deletion of only ORMDL3 could increase complex sphingolipids but not the sphingoid bases from which they are derived because of more efficient degradation.

## Discussion

ORMDLs negatively regulate de novo biosynthesis of ceramide by decreasing the activity of SPT (4, 5). Recent high-resolution cryo-electron microscopy structure of the SPT complex composed of catalytic components (SPTLC1 and SPTLC2) and regulatory component SPT small subunit A (ssSPTa) suggests that ORMDL3 is located in the center of the SPT complex, serving to stabilize the SPT assembly (35). Moreover, another cryo-EM study indicates ORMDL3 blocks the acyl-CoA binding tunnel and competes with substrate binding through its amino terminus (36). Although previously it was suggested that elevated free and phosphorylated sphingoid bases inhibit SPT via ORMDLs (37), more recent studies indicate that regulation of SPT by ORMDLs involves sensing of elevation in ceramide and its interactions with the membrane-bound components of the SPT regulatory apparatus (9). However, not much is still known about the role of ORMDLs, particularly ORMDL3 that has been implicated in asthma pathogenesis (10, 11, 12, 13), in regulating sphingolipid metabolism and levels of bioactive sphingolipid metabolites, and which specific sphingolipid levels are altered (14). As single knockdown of *ORMDL3*, but not *ORMDL1* or *ORMDL2*, increased levels of sphingolipids in cell culture ((23) and Fig. 1) and mice (18), we evaluated cells in which ORMDL3 alone or all three ORMDL proteins were deleted using CRISPR/Cas9 that has several advantages over siRNA approaches, including permanent and precise gene disruption with fewer off-target effects and lower risk of immune response (25, 33). Moreover, as KO of all three *Ormdls* is embryonic-lethal (18), ORMDL-TKO cells are very useful to examine whether individual ORMDLs have overlapping or redundant functions in regulating bioactive sphingolipids. In addition, they might be useful for the study of other potential inhibitors of SPT such as Nogo-B (38).

Here we show that complete deletion of ORMDL3 alone did not significantly affect SPT activity, yet a robust increase in SPT activity was observed in ORMDL-TKO cells. The substantial increase in de novo–derived dihydrosphingosine and dihydro-S1P is consistent with the dramatic increases observed in SPT activity and suggests that the three ORMDL isoforms are at least partially redundant in inhibiting SPT activity. Similarly, SPT activity was not changed in *ORMDL3* deleted or overexpressed cells (16) and it has been suggested that all three ORMDLs must be downregulated to relieve the inhibition of SPT (7). In fact, it was shown that complexes of ORMDLs normally interact with and regulate SPT in a stoichiometric manner that determines their effects on sphingolipid biosynthesis (6). The lack of significant effects of *ORMDL3* knockdown on measured SPT activity but clear effects on elevation of sphingolipid levels, both here and in previous studies (18, 23), is intriguing. This may indicate that SPT activity assays might not be sufficiently sensitive to measure small changes in its activity, whereas cumulative changes in sphingolipid metabolites can readily be measured by very sensitive MS. Alternatively, ORMDL3 and potentially the two paralogues regulate sphingolipid metabolism by mechanisms in addition to regulation of SPT.

Deletion of ORMDL3 alone slightly increased dihydroceramides and ceramides, consistent with previous studies of *Ormdl3* KO in mice (17, 18) and its downregulation in cells (21, 23, 39). Nevertheless, these increases did not lead to increased monoglucosylceramides but rather increased lactosylceramides and sphingomyelins. ORMDL-TKO cells had a much greater increase in levels of dihydroceramides and ceramides than ORMDL3-KO cells as expected if each of the three ORMDL isoforms contribute to inhibition of SPT. Because synthesis of GalCer occurs at the ER, it does not require ceramide transport. Thus, it is not surprising that a significant amount of the excess ceramides in the ER of the ORMDL-TKO cells is converted to GalCer. In contrast, ceramides generated at the ER must be transported by vesicular trafficking to the medial Golgi for the synthesis of GluCer. Subsequently, phosphatidylinositol-four-phosphate adaptor protein 2, via its GluCer transfer activity, promotes synthesis of lactosylceramides and complex glycolipids at the Golgi (2). Our observation of large increases in these glycosphingolipids species in ORMDL-TKO cells, predominantly in the very-long-chain species corresponding to the increased acyl chain ceramide species, suggests that an efficient vesicular transport system is maintained in these cells.

The ceramide transporter, CERT, which is selective for long-chain ceramides, is required for its transfer from the ER to the *trans*-Golgi for sphingomyelin synthesis (40). Surprisingly, although C16:0 is the major sphingomyelin species, it was only increased in ORMDL3-KO and not in ORMDL-TKO cells. Yet, all other N-acyl chain sphingomyelin species were increased in the triple KO cells. It is possible that at such high levels of ceramides, rates of their trafficking from the ER to the Golgi by CERT are saturated. Alternatively, if membrane contact sites between the ER and *trans* Golgi network in ORMDL-TKO cells are decreased, this would impede further transfer of ceramide by CERT, which is enriched at these membrane contact sites to prevent further increase in sphingomyelin biosynthesis.

Deletion of all three ORMDL isoforms also led to dramatic increases in the levels of dihydrosphingosine and dihydro-S1P. This suggests that the excess dihydrosphingosine that accumulates in the ER because of unregulated SPT is not only being acylated but also shunted toward phosphorylation and degradation by ER-localized SPL. Moreover, although sphingosine levels were only doubled, there was a large increase of S1P in ORMDL-TKO cells. The inherent preferential substrate specificity of the alkaline ceramidase ACER1 localized to the ER for very-long-chain ceramides that are predominantly increased in ORMDL-TKO cells suggests ACER1 may degrade these excess ceramides to sphingosine that is then converted to S1P in the absence of all ORMDL isoforms (3, 41). The increase in pro-survival S1P may also be a protective mechanism that enables ORMDL-TKO cells to survive the very high levels of pro-apoptotic ceramides. Together, our study provides a more comprehensive picture of ORMDL-mediated regulation of sphingolipid metabolism. These findings demonstrate ORMDL isoforms are only partially functionally redundant and indicate a potential role for ORMDLs in maintaining the complex, compartmentalized balance of de novo synthesis of sphingolipids as well as their degradation.

## Data availability

All data supporting this study are included in the manuscript and supplemental data.

## Supplemental data

This article contains supplemental data.

## Conflict of interest

The authors declare no conflicts of interest.
