# Supplementary figures and images for: CRISPR/Cas9 deletion of ORMDLs reveals complexity in sphingolipid metabolism
Source: J Lipid Res. 2021 Apr 30;62:100082. doi: 10.1016/j.jlr.2021.100082 (PMC8167824; doi:10.1016/j.jlr.2021.100082)

# Supplementary Figure 1.

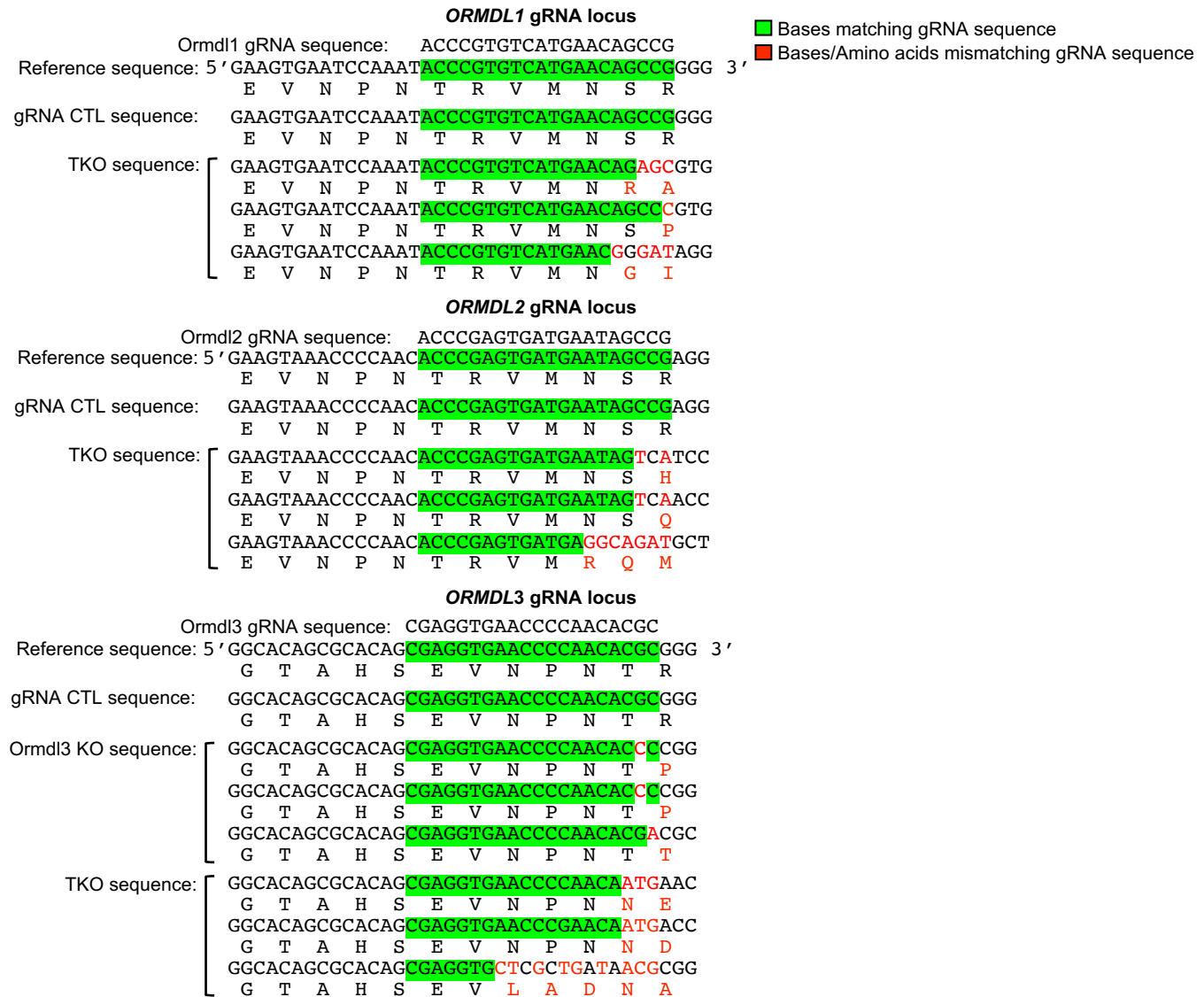

**Supplementary Figure 2.**

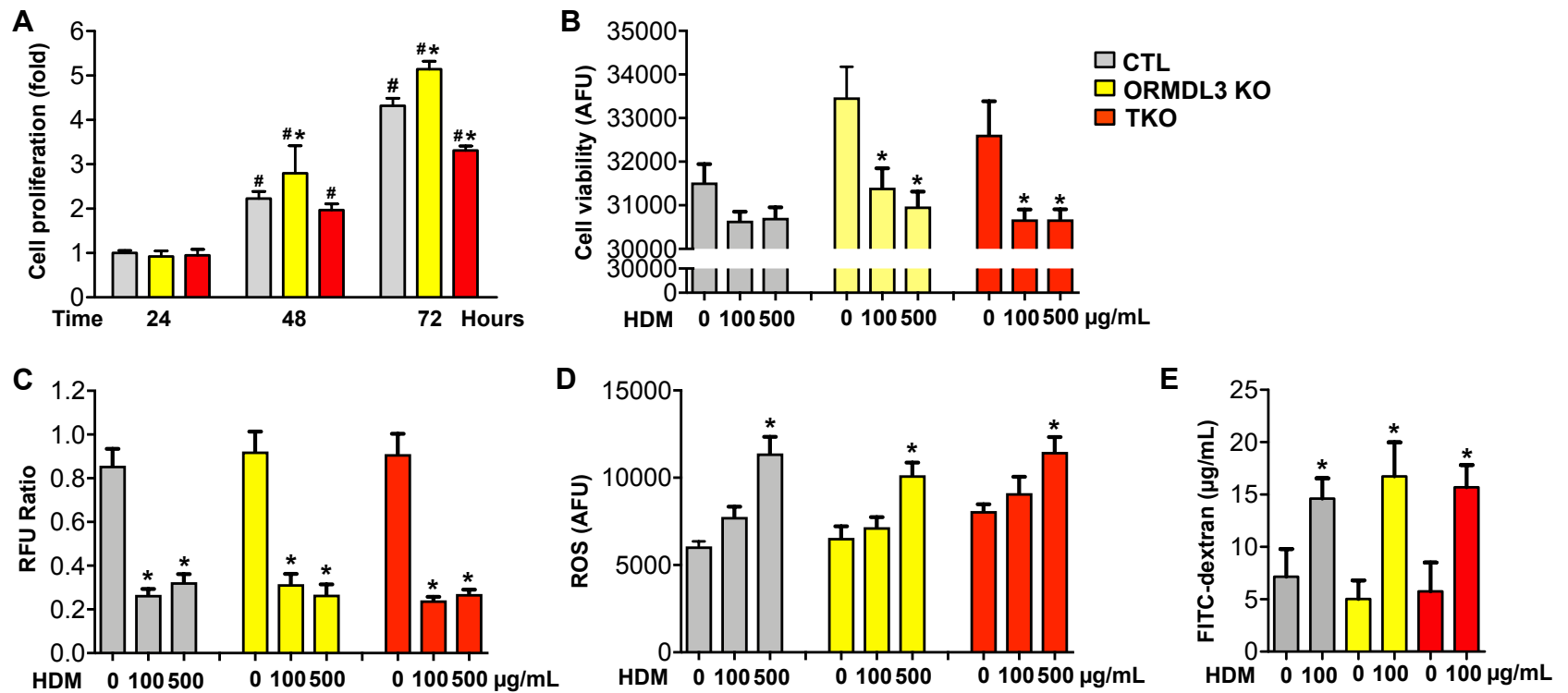

Supplement: Supplemental Figures S1 and S2 [file mmc1.pdf]
