# Supplementary material for: CRISPR/Cas9 deletion of ORMDLs reveals complexity in sphingolipid metabolism
Source: J Lipid Res. 2021 Apr 30;62:100082. doi: 10.1016/j.jlr.2021.100082 (PMC8167824; doi:10.1016/j.jlr.2021.100082)
Supplement: Supplemental Table S1 [file mmc2.pdf]

Supplementary Table 1. Detection of CRISPR/Cas9 editing with ORMDL sgRNAs by Sanger sequencing.

**CRISPR/Cas9 editing detection**						
**Primer locus**	**Target sequence**	**CTL1**	**CTL2**	**ORMDL3 KO1**	**ORMDL3 KO2**	**TKO**
ORMDL1 gRNA	ACCCGTGTCATGA ACAGCCG	No	No	No	No	Yes
ORMDL2 gRNA	ACCCGAGTGATGA ATAGCCG	No	No	No	No	Yes
ORMDL3 gRNA	CGAGGTGAACCCC AACACGC	No	No	Yes	Yes	Yes
ORMDL1 off-target 1	CCCCCTGTAATGA ACAGCCA	No	No	---	---	No
ORMDL1 off-target 2	TCCCTTGGCATGA ACAGCCT	No	No	---	---	No
ORMDL2 off-target 1	AACCGAGTGCTGA ATAGCAATGG	No	No	---	---	No
ORMDL2 off-target 2	ACCAAAGTGAGGA AGAGCCGAGG	No	No	---	---	No
ORMDL3 off-target 1	ATGGGTGAACCCC AACACAC	No	No	No	No	No
ORMDL3 off-target 2	CCTGCTGAACCCC TACACGC	No	No	No	No	No
ORMDL3 off-target 3	GGAGGTGGACCCC AACATCC	No	No	No	No	No
ORMDL3 off-target 4	GGAGGTGGACCCC AACATCC	No	No	No	No	No
ORMDL3 off-target 5	CCAGGTGAACACC AGCACCC	No	No	No	No	No
ORMDL3 off-target 6	GGAGGTGAACACC ACCACCC	No	No	No	No	No
ORMDL3 off-target 7	CGACGTGAACGAC AACGCGC	No	No	No	No	No
ORMDL3 off-target 8	CGACGTGAACGAC AACGCGC	No	No	No	No	No
ORMDL3 off-target 9	CGAGTTGCACCCC ACCACCC	No	No	No	No	No
ORMDL3 off-target 10	CGAGGTGCACCCC CACCCTC	No	No	No	No	No
ORMDL3 off-target 11	CGAGGTGAACCCG GACCCAC	No	No	No	No	No
ORMDL3 off-target 12	CGCGGTGCACCCC CAGACGC	No	No	No	No	No
