# Supplementary material for: CRISPR/Cas9 deletion of ORMDLs reveals complexity in sphingolipid metabolism
Source: J Lipid Res. 2021 Apr 30;62:100082. doi: 10.1016/j.jlr.2021.100082 (PMC8167824; doi:10.1016/j.jlr.2021.100082)
Supplement: Supplemental Table S2 [file mmc3.pdf]

Supplementary Table 2. Primer sequences for validation of CRISPR/Cas9 editing with *ORMDL* sgRNAs.

Primer locus	Target sequence	Direction	Primer Sequence
ORMDL1 gRNA	ACCCGTGTCATGAACAGCCG	Forward	AATTTCCGGCAACCACTAAAC
		Reverse	CTGCTGTCATCAGCATCTACA
ORMDL2 gRNA	ACCCGAGTGATGAATAGCCG	Forward	TCTGCGTGATGGGTAAGTTC
		Reverse	TACAAGGGCGTGGTTTAGTG
ORMDL3 gRNA	CGAGGTGAACCCCAACACGC	Forward	GTCAGTACATCACCCTGACAC
		Reverse	AGCAGCAGGAAATGAGTAGAG
ORMDL1 off-target 1	CCCCCTGTAATGAACAGCCA	Forward	CTGTGCTCAGCTCTCCTAATAC
		Reverse	GCCCTGGCTGTTCATTACA
ORMDL1 off-target 2	TCCCTTGGCATGAACAGCCT	Forward	AGCTCCCAGCTAAGAGAAGA
		Reverse	CTGGCACCCACTCTGATTT
ORMDL2 off-target 1	AACCGAGTGCTGAATAGCAATGG	Forward	ATGACCTGGGCTTGGATTG
		Reverse	GTCTACACGCACCACCTAAA
ORMDL2 off-target 2	ACCAAAGTGAGGAAGAGCCGAGG	Forward	GTTGGTGCTACAGTCCCTTTAT
		Reverse	GGAACACTGCTCCGAGATAAA
ORMDL3 off-target 1	ATGGGTGAACCCCAACACAC	Forward	TTCTGGGCTCAAAGTTCTAGTT
		Reverse	GGTCCTGGAGGCATTTGATTAT
ORMDL3 off-target 2	CCTGCTGAACCCCTACACGC	Forward	CTACAGGAGCTACGAGGATTTC
		Reverse	TACGACAAGGACAGCCATTC
ORMDL3 off-target 3	GGAGGTGGACCCCAACATCC	Forward	CCACTTGGTCTCAAGCATCT
		Reverse	TCTCCACTCCTTCTGGGATT
ORMDL3 off-target 4	GGAGGTGGACCCCAACATCC	Forward	GGGCTGGAGATGTGCATAG
		Reverse	AGAGCTCTCCTCACCAAGAA
ORMDL3 off-target 5	CCAGGTGAACACCAGCACCC	Forward	GTCCACCTGAAGACTGAGTATAG
		Reverse	CAGCAGGTTCAAGGTGCATA
ORMDL3 off-target 6	GGAGGTGAACACCACCACCC	Forward	CCTTTCCTGGCAGTACAATGA
		Reverse	CCTTGGATTTCGGCACATTTC
ORMDL3	CGACGTGAACGACAAC	Forward	CTCACTTCCCATCTCAGAGAAC

off-target 7	GCGC		
		Reverse	GGGTTGTTCTCCTTCACGAATA
ORMDL3 off-target 8	CGACGTGAACGACAAC GCGC	Forward	GGTGTCCACCTACAAGAACTAC
		Reverse	GAGCGTTGTCGTTCTCGT
ORMDL3 off-target 9	CGAGTTGCACCCCACC ACCC	Forward	AGTGCTGGGTCTTGAACAG
		Reverse	GTACTCCCTCCTCTCCACTC
ORMDL3 off-target 10	CGAGGTGCACCCCCAC CCTC	Forward	CTGTGATGTCGGTGGTGATG
		Reverse	AGGGCTTAGGGATCAGGAA
ORMDL3 off-target 11	CGAGGTGAACCCGGAC CCAC	Forward	GGAAGTCACAGGAACAGTTAGG
		Reverse	TGCTAAGGGAGAGCCTGTAA
ORMDL3 off-target 12	CGCGGTGCACCCCCAG ACGC	Forward	AGCCGTGTTTCCTTAGGATTG
		Reverse	GCCCTCCTGTACTTCTAAAGC
