# Supplementary material for: CRISPR/Cas9 deletion of ORMDLs reveals complexity in sphingolipid metabolism
Source: J Lipid Res. 2021 Apr 30;62:100082. doi: 10.1016/j.jlr.2021.100082 (PMC8167824; doi:10.1016/j.jlr.2021.100082)
Supplement: Supplemental Table S3 [file mmc4.pdf]

Supplementary Table 3. Fold change in sphingolipid species in ORMDL3 knockout cells

Sphingolipid	Cells	Sphingolipid species									
		**C14**	**C16:0**	**C18:1**	**C18:0**	**C20:0**	**C22:0**	**C24:1**	**C24:0**	**C26:1**	**C26:0**
Dihydroceramide	CTL	1.0±0.19	1.0±0.03	1.0±0.07	1.0±0.04	1.0±0.09	1.0±0.03	1.0±0.01	1.0±0.03	1.0±0.03	1.0±0.13
	ORMDL3 KO	1.0±0.22	2.9±0.48*	0.9±0.15	1.9±0.34#	2.0±0.50	1.9±0.41#	1.2±0.20	1.0±0.08	0.8±0.10	0.9±0.13
Ceramide	CTL	1.0±0.20	1.0±0.03	1.0±0.07	1.0±0.05	1.0±0.08	1.0±0.03	1.0±0.01	1.0±0.03	1.0±0.04	1.0±0.13
	ORMDL3 KO	1.0±0.22	2.9±0.48*	0.9±0.16	1.9±0.34#	2.0±0.51	1.9±0.41#	1.3±0.15	1.4±0.03**	0.8±0.09	0.9±0.12
Galactosyl-dihydroceramide	CTL	1.0±0.15	1.0±0.03	1.0±0.06	1.0±0.04	1.0±0.07	1.0±0.04	1.0±0.04	1.0±0.04	1.0±0.11	1.0±0.03
	ORMDL3 KO	1.1±0.30	0.8±0.07*	0.9±0.23	1.0±0.21	1.2±0.23	1.0±0.17	0.8±0.14	0.9±0.04	1.1±0.34	0.8±0.06#
Glactosylceramide	CTL	1.0±0.15	1.0±0.04	1.0±0.06	1.0±0.03	1.0±0.07	1.0±0.04	1.0±0.03	1.0±0.04	1.0±0.12	1.0±0.03
	ORMDL3 KO	1.1±0.28	0.8±0.07*	0.8±0.22	1.0±0.21	1.2±0.24	1.0±0.15	0.8±0.13	0.9±0.03	1.1±0.31	0.8±0.05*
Glucosyl-dihydroceramide	CTL	1.0±0.15	1.0±0.03	1.0±0.06	1.0±0.04	1.0±0.07	1.0±0.03	1.0±0.04	1.0±0.04	1.0±0.12	1.0±0.03
	ORMDL3 KO	1.1±0.29	0.8±0.07*	0.8±0.22	1.0±0.22	1.1±0.23	1.0±0.15	0.8±0.13	0.9±0.03	1.1±0.30	0.8±0.05*
Glucosylceramide	CTL	1.0±0.15	1.0±0.03	1.0±0.06	1.0±0.04	1.0±0.07	1.0±0.04	1.0±0.04	1.0±0.04	1.0±0.12	1.0±0.03
	ORMDL3 KO	1.1±0.28	0.8±0.07*	0.8±0.22	1.0±0.21	1.2±0.23	1.0±0.15	0.8±0.13	0.9±0.03	1.1±0.31	0.8±0.05*
Lactosylceramide	CTL	1.0±0.20	1.0±0.04	1.0±0.07	1.0±0.04	1.0±0.09	1.0±0.03	1.0±0.02	1.0±0.04	1.0±0.03	1.0±0.13
	ORMDL3 KO	0.9±0.22	2.8±0.51*	0.9±0.17	1.8±0.35#	1.9±0.50	1.8±0.42	1.2±0.17	1.3±0.06**	0.8±0.12	0.8±0.12
Monohexosyl-ceramide	CTL	1.0±0.15	1.0±0.04	1.0±0.06	1.0±0.04	1.0±0.07	1.0±0.04	1.0±0.05	1.0±0.04	1.0±0.12	1.0±0.16
	ORMDL3 KO	1.1±0.27	0.8±0.07*	0.8±0.22	1.0±0.21	1.2±0.23	1.0±0.15	1.0±0.15	0.9±0.02	1.1±0.31	1.5±0.37
Dihydro-sphingomyelin	CTL	1.0±0.07	1.0±0.02	1.0±0.06	1.0±0.02	1.0±0.04	1.0±0.03	1.0±0.05	1.0±0.01	1.0±0.05	1.0±0.09
	ORMDL3 KO	1.3±0.09#	1.3±0.03**	1.6±0.09**	2.4±0.13**	1.8±0.10**	1.5±0.04**	0.8±0.02*	1.2±0.04**	1.2±0.14	0.9±0.14
Sphingomyelin	CTL	1.0±0.06	1.0±0.02	1.0±0.06	1.0±0.06	1.0±0.05	1.0±0.03	1.0±0.04	1.0±0.01	1.0±0.05	1.0±0.09
	ORMDL3 KO	1.2±0.12	1.1±0.09**	1.2±0.09#	1.4±0.18	1.1±0.07	1.3±0.07**	1.1±0.05#	1.1±0.06#	0.9±0.07	1.0±0.09

# $p < 0.05$; * $p < 0.01$; ** $p < 0.001$ compared to CTL.
