# Supplementary material for: CRISPR/Cas9 deletion of ORMDLs reveals complexity in sphingolipid metabolism
Source: J Lipid Res. 2021 Apr 30;62:100082. doi: 10.1016/j.jlr.2021.100082 (PMC8167824; doi:10.1016/j.jlr.2021.100082)
Supplement: Supplemental Figure Legend [file mmc5.doc]

**Supplementary Figure 1. ORMDL expression and validation of CRISPR knockouts.** DNA sequencing of genomic gRNA target loci in CTL1, CTL2, ORMDL3 KO1, ORMDL3 KO2, and TKO cells. Data represent genomic DNA sequencing and translated amino acid sequences for each loci. DNA bases matching the gRNA sequence are in green. DNA bases or amino acids mismatching gRNA sequence are in red.

**Supplementary Figure 2. Effects of deletion of ORMDL1,2,3 on cellular responses to HDM.** (A) CTL, ORMDL3 KO, and TKO cells were cultured for the indicated days and proliferation determined with WST-8. Data represent mean ± SD (n=8-12). Each data point represents 2-3 independent experiments each with 4 separate biological replicates. ^#^ p < 0.001 compared to their respective 24 hr control by 1-way ANOVA with Tukey’s Post-Hoc analysis. ^*^ p < 0.05 compared to their respective CTL by 1-way ANOVA with Dunnett’s Post-Hoc analysis. (B-D) Cells were treated without or with the indicated concentrations of HDM for 24 h. (B) Cell viability was determined by Resazurin fluorescence measurements. (n=12-15). (C) Live/Dead ratio was determined by double staining with calcein-AM and PI. The ratio of fluorescence intensities of live and dead cells 485/535 nm to 530/620 nm, respectively, were expressed as relative fluorescence units (RFU) ratios (n=10-12). (D) Increased ROS in the cells were measured by oxidation of 2,7-dichlorofluorescein to fluorescent dichlorofluorescein. Data are means ± SDs (n=8). ^*^ p < 0.05 compared to their respective 0 μg/mL control by 1-way ANOVA with Dunnett’s Post-Hoc analysis. (E) Cells were cultured in Transwell inserts and treated without or with HDM as indicated. FITC-dextran was added to the upper chamber. Fluorescence intensity in the lower chamber was measured after 24 h. n=6, in 3 independent experiments. ^*^ p < 0.001 compared to their respective 0 μg/mL control by Student’s unpaired T-test.
